# Diffusion of a Peer-Led Suicide Preventive Intervention Through School-Based Student Peer and Adult Networks

**DOI:** 10.3389/fpsyt.2018.00598

**Published:** 2018-11-15

**Authors:** Trevor A. Pickering, Peter A. Wyman, Karen Schmeelk-Cone, Chelsey Hartley, Thomas W. Valente, Anthony R. Pisani, Kelly L. Rulison, Charles Hendricks Brown, Mark LoMurray

**Affiliations:** ^1^Department of Preventive Medicine, Keck School of Medicine, University of Southern California, Los Angeles, CA, United States; ^2^Department of Psychiatry, School of Medicine and Dentistry, University of Rochester, Rochester, NY, United States; ^3^Department of Public Health Education, University of North Carolina at Greensboro, Greensboro, NC, United States; ^4^Department of Psychiatry and Behavioral Sciences, Feinberg School of Medicine, Sources of Strength, Inc., Chicago, IL, United States

**Keywords:** suicide prevention, peer leaders, social networks, diffusion of innovations, social connectedness, school intervention, peer messaging, social support

## Abstract

**Background:** Peer-led interventions have been applied to prevent various health behavior problems and may be an important complement to individual-level suicide prevention approaches. Sources of Strength trains student “peer leaders” in secondary schools to conduct prevention activities that encourage other students to build healthy social bonds and strengthen help-seeking norms. Prior work examining diffusion of peer-led programs has focused on youths' closeness to peer leaders but minimally on other factors such as connections to adults and suicidal behavior.

**Methods:** We examined implementation and dissemination of Sources of Strength in 20 schools. Over 1 year 533 students were trained as peer leaders and 3,730 9th−12th graders completed baseline surveys assessing friendships and adults at school, and suicidal thoughts/behaviors; and end-of-year surveys reporting intervention exposure: viewed poster/video, attended presentation, direct peer communication, and activity participation. Chi-square tests compared exposure rates by student and network characteristics. Multi-level logistic regression models tested predictors of exposure across individual and school-level characteristics.

**Results:** Exposure to the intervention varied greatly by school and by individual student characteristics and network position. Training more peer leaders increased school-wide exposure for all modalities except presentation (Bs 0.06–0.10, *p's* < 0.05). In multivariate models, exposure was consistently higher for students closer to peer leaders in the friendship network (ORs 1.13–1.54, *p's* < 0.05) and students who named more trusted adults (ORs 1.08–1.16, *p's* < 0.001); and lower for males (ORs 0.56–0.83, *p's* < 0.05). In multivariate models, training more students as peer leaders predicted exposure to poster-video and direct peer communication in larger schools (OR = 3.34 and 2.87, respectively). Network characteristics influenced exposure similarly for students with suicidal thoughts and behaviors.

**Discussion:** Our findings confirm prior work showing the importance of personal affiliations to peer leaders and natural networks as a medium for diffusion of peer-led prevention efforts. We build on that work by showing independent effects of closeness to adults at school and number of peer leaders trained. There is a need to strategically select peer leaders to maximize closeness to students school-wide, particularly in larger schools. Additional work is required for Sources of Strength to devise messaging strategies to engage males and students isolated from adults at school.

## Introduction

School-based suicide prevention programs have utilized a narrow range of approaches along the continuum of public health interventions. Current programs focus on individual-level risk factors. The most widely used programs employ strategies—screening (1) and gatekeeper training (2)—to expand recognition and referral of adolescents at elevated risk for suicide due to psychiatric and/or behavioral problems (3). School-based educational programmes (4) are another approach designed to increase students' self-disclosure of suicide risk, identification of at-risk peers, and management of distress and depression symptoms (5–8). Despite widespread use of individual-focused interventions (9), youth suicide rates are increasing. Among those 15–19 years of age the U.S., suicide rates increased from 8.0 per 100,000 in 2000 to 9.76 per 100,000 in 2015, a 22% increase (10). Consensus is emerging among researchers and leading policy groups that a broader range of population-focused prevention approaches is needed to reduce suicide rates (11).

This study focuses on another type of public health preventive approach: preparing key opinion leaders to modify social-ecological protective factors, including healthy bonds to peers and adults, within their schools and social networks. Specifically, we examined implementation and dissemination of Sources of Strength, a peer-led suicide prevention program for secondary schools, typically encompassing individuals 14–18 years of age (12, 13). Our prior work identified Sources of Strength as the first intervention involving peer leaders to enhance protective factors associated with reduced suicidal behavior at the school population level (13). However, like nearly all other interventions employing key opinion leaders, minimal research attention has focused on identifying key implementation components of Sources of Strength and processes of dissemination through a population. To develop a more complete model for Sources of Strength, it is necessary to clarify the mechanisms behind intervention diffusion in a schoolwide context. Such knowledge is critical for determining how to maximize the intervention's efficiency and impact, especially considering that participating high schools may have widely different patterns of student social affiliations. We evaluated how characteristics of students' peer affiliation and adult networks influenced exposure to the Sources of Strength intervention messaging. A specific objective was to examine the extent to which messaging reached students who are isolated from peers and adults, as well as those at high risk for suicide due to suicidal thoughts and behaviors in the prior year.

### Key opinion leader interventions: evidence and research needs

Peer leaders' delivering prevention programming has been applied to a variety of adolescent health problems but only recently to school-based suicide prevention (13). School-based programs incorporating peer leaders and student-to-student exercises are more effective than lecture-style programs, according to meta-analyses of substance abuse interventions (14, 15). Peer leaders have been effective in reducing HIV risk behaviors (16–18), in other health promotion programs (19), and in tobacco use prevention (20–24). Variability in how peer-led programs are implemented is extensive. Peer-led tobacco prevention interventions, for example, range from preparing older students to deliver structured classroom lessons (23) to training influential students to encourage their peers not to smoke through informal interactions (20).

Adolescents influence their peers' prosocial and antisocial behaviors including drug use and health (25, 26), and the effectiveness of peer leaders is congruent with the fact that this influence occurs through individuals' social networks. Recent social network modeling indicates that many health behaviors, including smoking cessation and obesity, reflect person-to-person spread of behaviors within social clusters (27), and the proximity of individuals within a social network determines the degree to which they will influence each over time (28, 29). That evidence is also congruent with research showing that individuals closely tied through affiliation groups influence each other's adoption of new practices (30), and evidence regarding importance of peer norms on behavior (30–32). Regarding suicide risk, peer suicidal behavior may promote a norm that suicide is a common response to distress, and adolescents are more susceptible to suicide imitation than are other age groups (33).

Work on mechanisms of peer-led programs is limited. Several studies have examined closeness to trained peer leaders to elucidate how intervention effects diffuse through a population. For example, a study of a peer health advocate program showed that individuals in a drug using community who were closest to one of the trained peer health advocates were more likely to be exposed to the intervention and adopt risk avoidance strategies (34). Valente and colleagues found that adolescents benefitted more from a tobacco prevention curriculum the more closely they were affiliated in friendship groups to those adolescents who were peer leaders delivering the intervention (24). Stoebenau and Valente (35) showed that people in one Madagascar village had higher contraceptive knowledge and use when they were directly tied to community-based contraceptive deliverers. However, many questions remain unaddressed. Work is needed to identify factors that promote dissemination of different peer leader messaging strategies, which range from informal communication to structured presentations (36). Evidence that overall school social network structure influences diffusion of substance use prevention effects (37) also points to the need for more research examining school characteristics. To systematize school programs, work is also needed to identify how the proportion of a school population trained as peer leaders influences dissemination effects. A question of specific interest to Sources of Strength is how students' ties to adults influence their receptivity to peer leader messaging.

### Sources of strength

Sources of Strength recruits and trains key opinion leaders (i.e., peer leaders) along with school staff members as advisors. Peer leaders learn a model of health and resilience that emphasizes growing healthy social bonds (i.e., trusted adults, family support, positive friends) and resources to manage adversity, such as healthy activities and medical/mental health resources for suicide concerns. With ongoing adult mentoring, peer leaders conduct activities to disseminate “sources of strength,” with the aim of modifying peer norms regarding coping and help-seeking and increasing youth-adult connections, particularly among students isolated from adults. The rationale stems from evidence that suicidal behavior is lower among youth with stronger social bonds to family, peers and other adults; strong bonds may reduce suicide risk through protective mechanisms including enhanced psychological well-being (38), increased help-seeking (39), and normative social influences that encourage adaptive coping (13).

Our prior work testing Sources of Strength through a cluster randomized trial (18 high schools, 2,000 students) showed that within 3 months after adolescent peer leaders were trained and began implementing prevention messaging activities (i.e., presentations, posters, peer communication), norms about help-seeking and perceptions of adult support were changed among students throughout their schools (13). However, this study employed traditional survey methods in which social network information was not collected. In a subsequent study we contrasted peer leader messaging activities (36 classrooms; 700 students) (36) and showed that peer leader presentations based on modeling of healthy coping increased other students' positive coping attitudes and perceptions of adult support; the addition of student audience involvement in identifying their own trusted adults increased students' expectations of adult support (36). Our findings suggest that peer leaders' involving classmates in interactive “sources of strength” messaging will increase impact of their messaging, congruent with communication theories emphasizing personal engagement (40).

### Current study

The current study primarily aimed to examine the diffusion of peer leaders' messaging activities across the student populations of 20 high schools over one school year, as part of a larger randomized controlled trial of Sources of Strength (clinicaltrials.gov #02043093). We sought to use students' nominations of friends at school (collected prior to training peer leaders) to identify individual student's network position, as well as overall school network properties. Students' nominations of their trusted adults at school helped inform the presence of youth-adult networks. The principal outcome of interest was exposure to Sources of Strength messaging modalities along a continuum of engagement that ranged from viewing posters/videos to participation in interactive exercises (e.g., naming a trusted adult).

We expected that peer leaders would reach more students who had closer friendship ties to peer leaders and more ties to other students overall (i.e., higher “centrality”). In contrast, we expected that students with fewer direct ties to peer leaders and to other students would have fewer opportunities for exposure to Sources of Strength. Prior work by Valente and colleagues showed that (a) individuals with exposure to external influences were critical in the diffusion of innovative practices, and (b) although external exposure played a role in bringing innovations to individuals' attention, the interpersonal persuasion of trusted others was crucial in convincing individuals to adopt (41–43). Thus, Valente's social network threshold model would predict that having social ties facilitates exposure to influences such as Sources of Strength messages, and close ties to peer leaders may be necessary for students to become more deeply involved such as participating in a Sources of Strength activity.

At the school level, we sought to examine the proportion of students trained as peer leaders, as well as characteristics of the school-wide peer network. A finding that schools with denser friendship networks had overall greater exposure to Sources of Strength among their students would be consistent with the network thresholds model that emphasizes diffusion through natural networks. We expected that the impact of school-level factors on exposure would be greater in schools with more students, since larger networks tend to be more fragmented.

A final set of questions focused on determining the extent to which peer leader messaging reached suicidal students and students who are isolated from peers and adults at school. In other analyses examining these school networks, we found that students with recent suicide attempts were more likely to be part of affiliation groups that were less cohesive and on the periphery of the school network (44), which are network positions that may reduce opportunities for exposure to Sources of Strength messaging. We were also interested if exposure varied by student sex. In our prior examination of peer-led classroom messaging, we found greater benefit for females vs. males in terms of perceptions that adults are capable of helping suicidal youth (36).

## Methods

### Schools and student enrollment

The 20 schools in this study were part of an effectiveness trial of Sources of Strength involving a total of 40 high schools located in predominantly rural, small town, and micropolitan communities of New York State and North Dakota. Schools in both states were recruited from counties or public health regions with past 5-year youth suicide rates above the state average (24.40 per 100,000 in North Dakota and 5.19 in New York for youth 15–19 in 2009–2011) (45). The 40 high schools were enrolled in four cohorts (2010–2013), with schools stratified by size and location; matched pairs were subsequently randomized into condition. The 20 high schools randomized to begin immediate implementation of Sources of Strength are included in this study (16 in New York, four in North Dakota). The schools ranged in size from 63 to 1,207 students (*M* = 366). Two schools served American Indian reservations.

Student recruitment occurred in two phases: (a) in early fall for participants in school-wide evaluation of Sources of Strength, and (b) immediately following for student peer leaders. For the school-wide assessments, all 9–12th graders were invited to enroll in the study evaluating Sources of Strength by completing fall and spring web-based assessments over two school years; a small portion (<1%) without language ability to independently complete web assessments were excluded. Information letters sent to parents included an option to decline their child's participation. This study was carried out in accordance with the University of Rochester Institutional Review Board who approved the study protocol. Information letters were sent to parents that included an option to decline participation. Research personnel collected opt-out forms and conducted verbal assent with eligible students followed immediately by web-based assessments. All students received information about how to access help or support for themselves or a peer if needed.

### Sources of strength intervention

Implementation of Sources of Strength in each school followed three standardized phases: (1) School community preparation, including training several staff members as advisors; (2) Recruitment and training of student peer leaders (PLs); and (3) peer leader messaging. Schools did not begin peer leader recruitment and training until baseline assessments of the student population were completed. Adult advisors facilitated standard recruitment procedures by distributing nomination forms school-wide and each staff member was asked to nominate up to 6 students whose “voices are heard” by other students. Nominations were reviewed to invite 5–10% of the student population across diverse groups within the school. Given 5–10% of the school population were invited, peer leader teams were dependent on school size. Of the 959 invited (19–86 per school), 798 (83.2%) enrolled with parent permission and youth assent/consent across two school years.

The training for peer leaders (along with their adult advisors) emphasized interactive learning about eight protective “sources of strength” (family support, positive friends, mentors, healthy activities, generosity, spirituality, medical access, and mental health access). Each school received half-day training [standardized curriculum of 15 modules (12) led by the program developer, co-author ML]. In the training peer leaders learned skills to increase protective resources in themselves, encourage peers to grow these resources, and connect suicidal peers with resources, especially trusted adults.

During the messaging phase, adult advisors led peer leader team meetings (bi-weekly to monthly) where they fostered the 8 protective strengths, built community within the peer leaders, and planned messaging activities for dissemination. The curriculum included student activities aimed at raising awareness of Sources of Strength, generating conversations with peers, providing presentations for peer leaders to share personal examples of using strengths, and engaging other students to identify their own trusted adults. Peer leaders were encouraged to tailor the messaging style to their school, with adult advisor monitoring to ensure safe messaging.

### Measures

Students completed questionnaires measuring: (a) suicidal thoughts and behaviors and (b) social networks, in the fall before peer leader training (baseline, Time 1). Students completed a questionnaire covering several modalities of exposure to Sources of Strength at the end of the school year (Time 2).

#### Suicidal behaviors

Suicidal ideation and attempts were assessed using the Youth Risk Behavior Survey measure (46) that has well-established reliability and validity for population-based assessments (47, 48). Each student was asked whether in the preceding 12 months she/he had: seriously considered suicide; planned suicide; made one or more suicide attempts; and made an attempt that resulted in medical injury requiring treatment. Suicide attempt was classified as having one or more attempts regardless of injury or ideation. Suicide ideation-only was classified as having suicide ideation with no suicide attempts.

#### Network measures

Students were asked to name up to seven of their closest friends at school, a peer network standard. Students nominated friends by typing in their names, a nomination method that yields fewer, closer relationships compared to selecting friends from a roster of names (49). A novel aspect of the network assessment was that students were also asked to name up to seven “adults in your school who you trust and feel you can talk to about personal things” (trusted adults).

Variables measuring the centrality of students in the network and closeness to peer leaders included: (a) Out-degree: the number of friends the student named. (b) Peer isolate: Students who named no friends and received no friendship nominations from others. (c) Coreness: An individual's coreness value *k* is the largest value that satisfies the following condition: the individual has at least *k* friends who also have at least *k* friends. For example, if a student is connected to 4 friends who each has at least 4 friends, that student would have a coreness value of 4. Coreness therefore reflects the extent to which an individual is a part of an interconnected friendship group and indicates the size of that group. (d) Closeness to peer leader: the number of steps in the shortest path to a peer leader, categorized into 1, 2, 3 or more steps, and not connected (i.e., there was no friendship path that connected to a peer leader). (d) Adult out-degree: total number of trusted adults named. Students who named no trusted adults were considered adult isolates.

Variables at the network level were normalized across 20 schools to have a mean of 0 and standard deviation of 1. These variables included: (a) School size: total school enrollment at Time 1. For analyses, school size was log-transformed to better fit a normal distribution. (b) School percent peer leaders: percent of school trained as a peer leader. (c) School scaled density: percent of total nominations made out of total nominations possible (maximum of 7 per student, or 7N for a school of size N).

#### Intervention exposure

Exposure to Sources of Strength was categorized into four different dichotomous exposure modalities corresponding to various levels of engagement. The student survey contained a section asking about exposures, preceded by the phrase, “Some students in your school have been trained as Peer Leaders in a program called Sources of Strength.” Students were subsequently asked about:

1. Exposure to a *presentation or assembly* consisted of answering “yes” to either: Have you seen a presentation or assembly about… (a) strengths that help teens get through hard times?, or (b) helping suicidal teens by getting adults involved? Example presentations included peer leaders leading presentations in their class about the “Sources of Strength wheel” and a source they felt they were strong in.
2. Exposure to *posters or videos* was assessed by answering “yes” to: Have you seen posters or videos at school about strengths? Example posters included pictures of the Sources of Strength wheel displaying the eight different sources.
3. *Direct peer communication* was measured by answering “yes” to either: Has a friend or other student… (a) told you about Sources of Strength?, or (b) talked to you about using strengths?
4. Participation in an *activity* consisted of answering “yes” to either: (a) Have you participated in a Sources of Strength activity such as adding your trusted adult to a poster?, or (b) Has a friend or other student asked you to name adults you can go to for help?

### Statistical analysis

Descriptive analyses were conducted in SPSS (V23). Creation and analyses of network variables were conducted in R v3.4.2 (50) with the igraph package (51). Gephi v0.9.2 (52) was used to graph network diagrams. Chi-square tests of proportions were used to determine if suicide ideation-only, suicide attempt, peer isolation, and adult isolation varied by demographic characteristics. This approach was also used to determine if demographic characteristics, suicidality, or network variables were associated with the four exposure modalities to Sources of Strength.

To examine the simultaneous effect of hypothesized individual- and school-level influences on Sources of Strength exposures, we fit multi-level logistic regression models using the lme4 package in R (53). These models included level-1 variables (sex, ethnicity, out-degree, coreness, closeness to a peer leader, trusted adults, suicide attempt, suicide ideation-only), level-2 variables (schoolwide density, schoolwide percent students trained as peer leaders, and school size), and a random intercept for school. To determine if the effect of closeness to a peer leader on exposure was moderated by having a more cohesive friendship group, an interaction between coreness and closeness to peer leaders was included. Similarly, an interaction between school size and school percent peer leaders was included as we hypothesized the effect of percent peer leaders would be greater in larger schools. We tested the significance of these interaction terms in each model and if the interaction was non-significant (*p* > 0.05) then it was excluded from the model.

Lastly, we examined if having suicide attempt or ideation moderated the effects of network variables on exposure. After evaluating each of the previous models, an interaction term was added for each predictor with (1) suicide attempt and (2) suicide ideation, individually. Predictors that had a significant interaction with either suicide attempt or suicide ideation-only were retained in the model.

## Results

### Sample participation and demographics

#### Student participants in school-wide assessments

Across the 20 schools, average school population enrollment was 82.2% (range 65.9–98.3%). A total of 5,677 students completed the assessments (baseline; Time 1) before training of student peer leaders (see Table 1). Participants included roughly equivalent proportion of males and females and grade levels. The enrolled sample was predominantly white and non-Hispanic, consistent with the rural and micropolitan communities of New York State and North Dakota. In the prior 12 months, 7.0% had made one or more suicide attempts (see Table 1, column 2) and 8.4% had suicidal ideation without attempt (see Table 1, column 3). Females had higher rates of suicide attempts and ideation, consistent with national norms (54). On the social network assessment, 194 students (3.4%) were peer isolates (i.e., students who neither made nor received a friendship nomination; see Table 1, column 4). A total of 2,082 students (36.1%) did not name a trusted adult at their school (see Table 1, column 5). Males were more likely to be isolated from peers and adults at school. More black/African American and other race students were peer isolates compared to white students. All minority race/ethnic groups were significantly more isolated from adults vs. white, non-Hispanic youth.

**Table 1:** Characteristics of students participating in school-wide assessments at Time 1.

		***N* (%)**	**Suicide attempt *N* (%)**	**Suicidal ideation *N* (%)**	**Isolate from friends *N* (%)**	**Isolate from adults *N* (%)**
Total		5,677	397 (7.0)	502 (8.8)	194 (3.4)	2,082 (36.1)
Sex	Male^†^	2,874 (49.9)	139 (4.8)	172 (6.0)	116 (4.0)	1,213 (42.3)
	Female	2,803 (48.6)	278 (9.9)*	325 (11.7)*	73 (2.6)*	829 (29.6)*
Grade	9th^†^	1,486 (25.8)	121 (8.4)*	118 (8.0)	39 (2.6)	629 (42.4)*
	10th	1,501 (26.0)	115 (8.0)	135 (9.1)	52 (3.5)	552 (36.8)
	11th	1,315 (22.8)	93 (7.3)	125 (9.6)	46 (3.5)	454 (34.6)
	12th	1,306 (22.7)	81 (6.4)	112 (8.7)	48 (3.7)	380 (29.2)
Race	Asian	133 (2.3)	9 (7.2)	14 (10.6)	3 (2.3)	65 (48.9)*
	Black/AA	588 (10.2)	47 (8.3)	34 (5.9)*	35 (6.0)*	268 (45.8)*
	Am. Indian	270 (4.7)	26 (9.9)+	17 (6.3)	7 (2.6)	117 (43.3)*
	White^†^	4,248 (73.7)	285 (6.9)	390 (9.5)	117 (2.8)	1,347 (31.8)
	Other	408 (7.1)	40 (10.4)*	37 (9.1)	22 (5.4)*	218 (53.6)*
Ethnicity	Hispanic^†^	503 (8.7)	61 (12.9)	54 (10.9)	27 (5.4)	268 (53.5)
	Non-Hisp.	5,147 (89.3)	357 (7.2)*	443 (8.7)+	159 (3.1)*	1,761 (34.3)*

* *p < 0.05*.

+ *p < 0.10, for difference in proportions between/among groups*.

† *Reference group. Categories may not add to 100% due to missing data*.

Among the enrolled non-peer leader students, 70.4% (*n* = 3,730) participated again at Time 2 and provided data on their exposure to Sources of Strength messaging. We examined if students who participated at both Time 1 and Time 2 were different from those who only participated at Time 1 (i.e., differential attrition). The groups were comparable by student sex and presence of suicidal ideation. However, white students were more likely to participate in both assessments vs. all minority race students. There were also fewer suicide attempts among those with both surveys (6.4%) than those with only Time 1 data (10.8%). Those who did not take the Time 2 survey were more likely to be isolated from peers vs. those who took both surveys (4.8 vs. 2.1%, respectively) and isolated from adults (44.8 vs. 31.8%). An additional 711 students participated at Time 2 only but were not included in analyses for this study.

#### Student peer leaders

**Figure 1:**
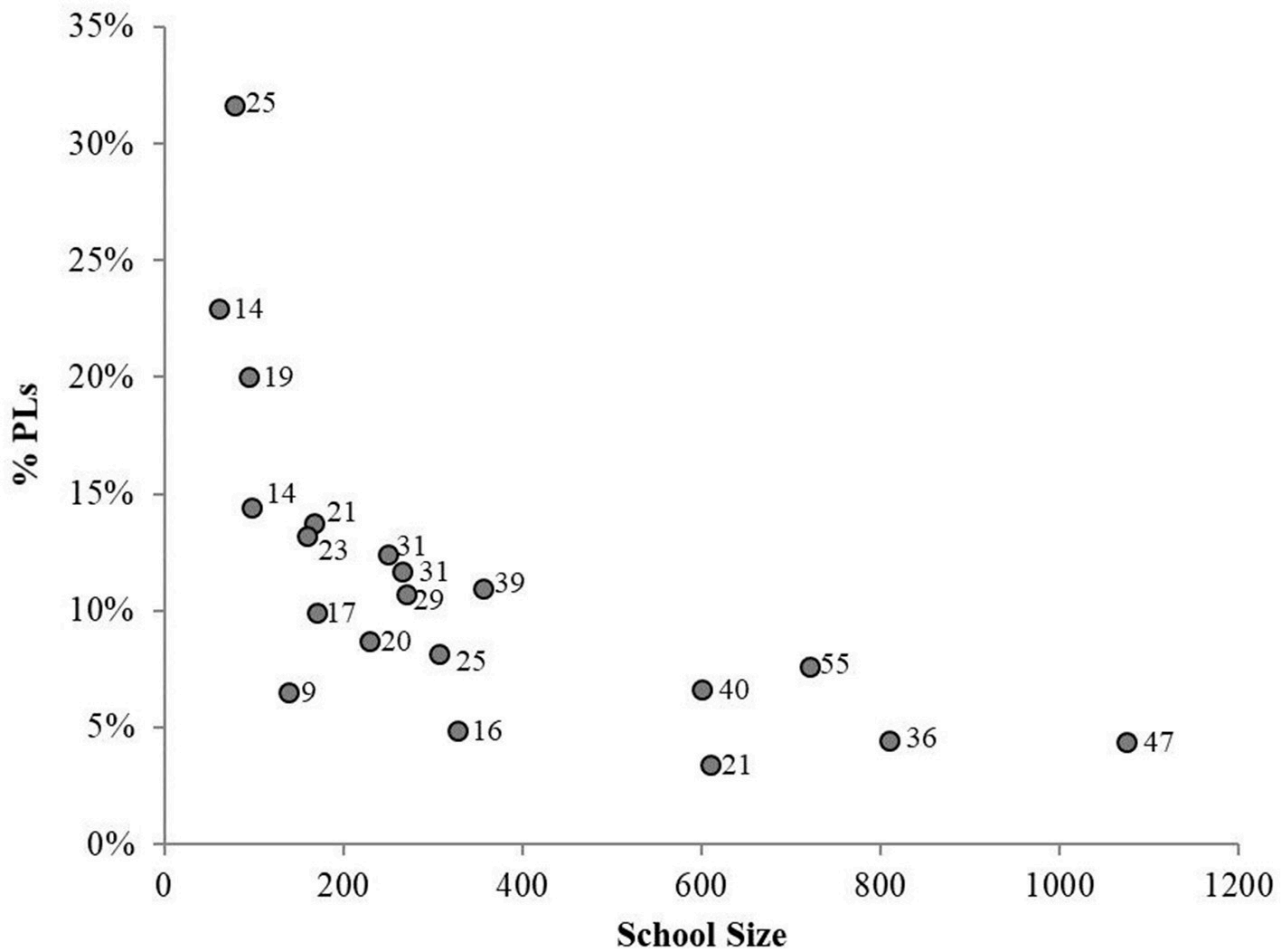
Percent of school trained as a peer leader by school size. Nodes are labeled with total number of peer leaders.

A total of 533 students enrolled and trained as peer leaders across the 20 schools (range 9–55 per school; see Figure 1, nodes labeled total PLs). The mean percent of total students trained as peer leaders was 9.2% (range 3–32%). The percent of students trained was sharply higher for smaller vs. larger schools, as shown in Figure 1. Schools with fewer than 200 students generally trained between 15 and 30% of students. Schools with more than 400 students did not train more than 10%. Peer leaders were made up of more females (56.5%). Regarding race, peer leaders had fewer black/African American members (3.7%) than non-peer leaders (5.8%); otherwise the groups were similar by race. Across grades, peer leaders had more 10th (28.5%) and 11th graders (30.1%) vs. non-peer leaders (27.2 and 23.3%, respectively). Peer leaders and non-peer leaders were similar in likelihood of reporting suicide attempts and suicidal ideation.

### Exposure to sources of strength by student and school characteristics

Overall, exposure to the intervention varied by messaging modality (see Table 2), i.e., 48.7 (activity participation) to 57.9% (poster/video exposure). Females consistently had greater exposure to the intervention across all modalities (*p's* < 0.001). Students with suicide attempt were less likely than those with no suicidal thoughts/behaviors (STB) to have seen a presentation or a poster/video (*p's* < 0.05). Having a friend who was a peer leader systematically led to higher exposure rates across all modalities (*p's* < 0.01). Students who named any friend at the beginning of the year had higher exposure rates for all modalities except participation in a Sources of Strength activity (*p's* < 0.05). Being isolated from adults was detrimental to exposure; being an isolate from adults consistently resulted in lower exposure rates across all modalities (*p's* < 0.001).

**Table 2:** Sources of Strength exposure by modality for non-peer leader students after one school year.

**Group**		***N***	**Poster/video (%)**	**Presentation (%)**	**Direct peer (%)**	**Activity (%)**
Total Non-PL		3,730	57.9	51.6	56.6	48.7
**DEMOGRAPHICS**						
Sex	Males^†^	1,908	51.2	48.9	49.9	43.5
	Females	1,778	64.9*	54.5*	63.8*	54.4*
Grade	9th	993	57.5	54.1*	57.7*	49.7
	10th	990	60.4	53.7*	61.1*	51.4*
	11th	848	55.2	50.7	55.8*	47.1
	12th^†^	809	58.0	48.0	50.8	45.9
STB	None^†^	3,105	58.5	52.5	56.8	49.4
	Ideation	310	56.4	52.7	58.2	49.7
	Attempt	235	49.6*	45.3*	56.4	45.7
**SOCIAL NETWORK**						
PL proximity	PL friend^†^	1,528	68.0	56.0	68.6	54.0
	No PL friend	2,194	52.7*	51.0*	50.4*	47.2*
Peer	Isolate^†^	80	38.8	35.0	43.8	40.0
	Non-isolate	3,642	58.3*	52.0*	56.9*	48.9
Adult	Isolate^†^	1,240	48.1	44.6	46.5	40.8
	Non-isolate	2,483	62.7*	55.1*	61.7*	52.7*
**SCHOOL-LEVEL**						
Mean of schools	20	63.0	52.7	59.3	51.3	
Range		23.4–85.1	30.7–84.3	20.3–76.5	25.0–83.0	
School size	Sm (< 150)^†^	8	69.8	55.5	61.8	62.5
	Md (150–500)	7	66.5	50.7	62.8	41.4*
	Lg (500+)	5	47.3*	50.9	50.4*	47.2

*STB, suicidal thoughts/behaviors. Subgroup sample sizes may not equal total sample size due to missing data*.

† Reference group

* *p < 0.05 for difference in proportions*.

**Figure 2:**
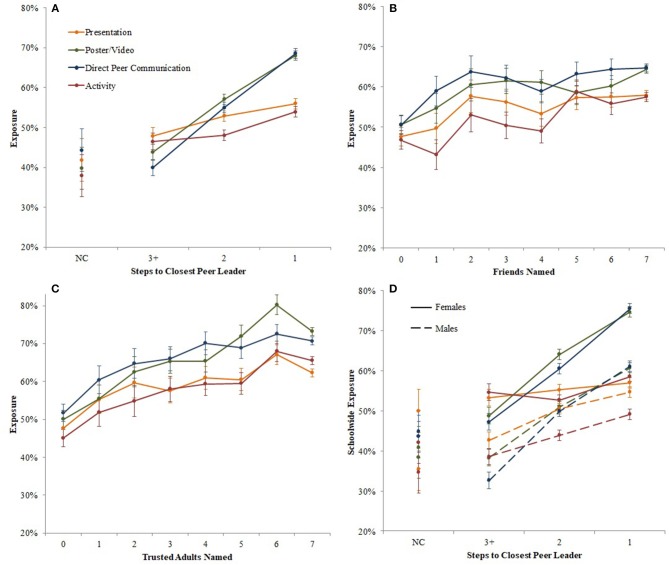
School-wide intervention exposure by **(A)** steps to closest peer leader, **(B)** number of friends named, **(C)** number of trusted adults named, and **(D)** steps to closest peer leader, stratified by gender (female = solid lines, male = dashed lines). Error bars represent one standard error.

Exposure was greater when students were closer to peer leaders and named more friends and trusted adults (i.e., an exposure-response relationship). That is, individuals who were closer in steps to a peer leader had even greater likelihood of exposure (Figure 2A). This effect was most pronounced for direct peer communication and poster/video (OR = 1.80 and 1.65, respectively, *p's* < 0.001) than for presentation and activity (OR = 1.16 and 1.19, respectively, *p's* < 0.001). For example, direct peer communication exposure for students who named a peer leader friend was 68.6%, decreased to 55% for having a peer leader as a friend of a friend, and 40% for being three or more steps away. Likewise, naming more friends modestly and incrementally increased likelihood of exposure for each additional friend named (Figure 2B; ORs ranged across exposure modalities from 1.05 to 1.07 per friend named, *p's* < 0.001) and naming more trusted adults increased the likelihood of exposure for each additional adult named (Figure 2C; ORs ranged across exposure modalities from 1.10 to 1.18 per adult named, *p's* < 0.001).

To determine if the lower exposure rates for males was explained by fewer friendship ties between males and peer leaders, we stratified this analysis by gender (Figure 2D). The relationship between exposure and closeness to peer leaders was similar for males and females, although uniformly lower for males. And males had lower exposure even when they named a peer leader friend. For example, direct peer communication exposure for females and males who named a peer leader as a friend was 76 and 61%, respectively.

**Figure 3:**
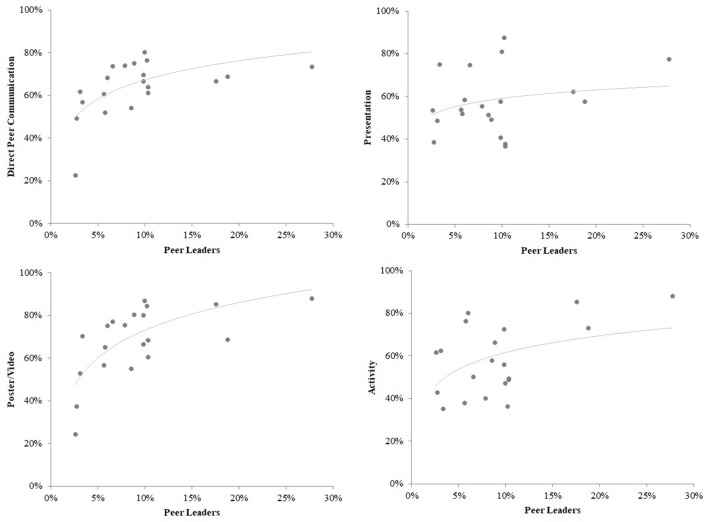
Exposure to the Sources of Strength intervention by percent peer leaders at school.

Intervention exposure varied greatly among schools. The range of schoolwide exposure proportion was 23.4–85.1% for poster/video, 30.7–84.3% for presentation, 25.0–83.0% for direct peer communication and 20.3–76.5% for activity (see Table 2). Exposure to the intervention trended lower in larger schools, though this effect reached traditional significance levels only for poster/video exposure in large schools vs. small schools and for presentation exposure in medium vs. small schools (*p's* < 0.05). Schoolwide percent of students trained as a peer leader was generally associated with exposure (Figure 3). A linear regression showed significant relationships between percent of students trained as peer leaders and having seen a poster/video (*B* = 1.57, *p* = 0.008), participating in an activity (*B* = 1.47, *p* = 0.01), and having direct peer communication (*B* = 0.98, *p* = 0.04), but not having seen a presentation (*B* = 0.70, *p* = 0.22).

Correlations of school-wide and individual-level variables are presented in Table 3. At the school level, students' mean closeness to a peer leader was related to several variables including intervention exposures (all modalities except having seen a presentation), percent of students trained as peer leaders, school size, and school density. Schoolwide suicide attempt and ideation-only rates were not correlated with any other variables at the school level. At the individual level, network characteristics were modestly related among each other and were all related to an individual intervention exposure across all modalities. Suicide attempt was inversely related to individual network centrality measures such as out-degree, coreness, and closeness to a peer leader. Number of trusted adults named was positively correlated with all individual exposure measures and all individual network centrality measures, and inversely related to suicide attempt and ideation-only.

**Table 3a:** Correlations among network and intervention exposure variables.

**(A) Level-2 (schoolwide) variables** (***N*** = **20)**								
**S.no**	**Measure**	**1**	**2**	**3**	**4**	**5**	**6**	**7**
1	Presentation	–						
2	Poster/video	**0.57**	–					
3	Direct peer com.	0.40	**0.88**	–				
4	Activity	−0.03	0.22	0.01	–			
5	% PLs	0.29	**0.58**	**0.47**	**0.54**	–		
6	Size	−0.28	–**0.63**	−0.40	–**0.55**	–**0.73**	–	
7	Density	0.05	0.24	0.23	0.30	**0.70**	−0.26	–
8	Mean closeness to PL	0.27	**0.72**	**0.60**	**0.49**	**0.92**	–**0.80**	**0.67**
9	Trusted adults	0.12	0.33	0.35	0.05	**0.43**	−0.20	**0.66**
10	Suicide attempt	0.00	−0.13	−0.10	−0.16	−0.25	0.19	−0.40
11	Ideation-only	−0.13	0.10	0.11	−0.24	0.02	−0.12	0.02

*Variables in bold are significant at p < 0.05. Variables 8–11 are individual-level metrics aggregated at the school level*.

**Table 3b:** 

**(B) Level-1 (individual) variables (*****N*** = **3,621–5,746)**											
**S.no**	**Measure**	**1**	**2**	**3**	**4**	**5**	**6**	**7**	**8**	**9**
1	Presentation^‡^	–								
2	Poster/video^‡^	**0.39**	–							
3	Direct peer^‡^	**0.30**	**0.44**	–						
4	Activity^‡^	**0.50**	**0.35**	**0.33**	–					
5	Out-degree	**0.06**	**0.08**	**0.09**	**0.09**	–				
6	Coreness	**0.05**	**0.11**	**0.10**	**0.08**	**0.70**	–			
7	Closeness to PL	**0.06**	**0.18**	**0.20**	**0.07**	**0.44**	**0.60**	–		
8	Trusted adults	**0.11**	**0.14**	**0.18**	**0.14**	**0.45**	**0.36**	**0.27**	–	
9	Suicide attempt^‡^	−**0.03**	−**0.05**	**–**0.01	**–**0.02	−**0.09**	−**0.11**	−**0.04**	−**0.07**	–
10	Suicide ideation^‡^	0.00	0.01	0.00	**–**0.01	−**0.04**	−**0.04**	**–**0.01	**–**0.03	−**0.08**

‡ *Dichotomous variable; the correlation coefficient displayed is the point-biserial correlation. Variables in bold are significant at p < 0.05*.

### Multivariate analysis

The multi-level logistic regression model indicated substantial variability in random intercept among schools (random intercept SD ranged from 0.44 to 0.62 by exposure modality), reflecting the differing school-wide exposure rates (Table 4a). Male gender was associated with lower exposure across all modalities (Table 4b; ORs ranged from 0.83 to 0.56, *p's* < 0.05). Two network variables were consistently associated with higher likelihood of intervention exposure: closeness to a peer leader and number of trusted adults named. Even when adjusting for all other individual-level network metrics, naming more trusted adults increased exposure to the intervention (ORs ranged from 1.08 to 1.16, *p's* < 0.001). Students with suicide attempt were less likely to have seen a poster/video (OR = 0.69, *p* < 0.001) and were less likely to have seen a presentation (OR = 0.76, *p* = 0.07). Ego's coreness moderated the effect of closeness to a peer leader on exposure to peer communication (interaction logit = 0.05, *p* < 0.05). That is, the effect of being close to a peer leader on peer communication was even stronger if the student was a part of a more dense, cohesive friendship group (see Table 4b; ORs ranged from 1.40 for low coreness to 1.68 for high coreness, *p's* < 0.05).

**Table 4a:** Logit coefficients and standard errors (A) and odds-ratios (B) from a multi-level logistic regression model in 20 schools.

**(A) Logit parameter estimates and standard errors**					
		**Exposure modality**			
	**M (SD)**	**Presentation**	**Poster/Video**	**Peer comm**.	**Activity**
Analytic Sample Size		3,418	3,445	3,439	3,425
Intercept		−0.20 (0.31)	−0.15 (0.29)	−0.74 (0.29)	0.08 (0.30)
**LEVEL-1 (INDIVIDUAL)**					
Gender (Male v. Female)	50.6%	−0.19 (0.07)*	−0.57 (0.08)**	−0.58 (0.08)**	−0.42 (0.07)**
Ethnicity (White v. Nonwhite)	72.1%	−0.16 (0.10)	0.01 (0.11)	−0.03 (0.11)	−0.01 (0.10)
Out-Degree	4.8 (2.7)	0.03 (0.02)	0.01 (0.02)	−0.04 (0.02)+	0.01 (0.02)
Coreness	5.0 (0.7)	−0.02 (0.03)	0.03 (0.03)	−0.08 (0.05)	−0.04 (0.03)
Closeness to PL	2.8 (0.8)	0.13 (0.06)*	0.22 (0.06)**	0.43 (0.06)**	0.14 (0.06)*
Trusted Adults	2.3 (2.4)	0.08 (0.02)**	0.09 (0.02)**	0.15 (0.02)**	0.10 (0.02)**
Suicide Attempt	7.61%	−0.27 (0.15)+	−0.36 (0.15)*	−0.02 (0.15)	−0.17 (0.15)
Suicide Ideation	8.81%	−0.10 (0.14)	−0.07 (0.15)	−0.06 (0.15)	−0.09 (0.15)
Coreness x Closeness to PL		-	-	0.05 (0.02)*	–
**LEVEL-2 (SCHOOL)**					
Density	0.60 (0.09)	−0.19 (0.18)	−0.22 (0.17)	−0.27 (0.15) +	0.17 (0.18)
Percent PLs	9.2% (6.1%)	0.17 (0.28)	0.77 (0.37)*	0.68 (0.32)*	−0.57 (0.38)
Size	5.3 (0.8)	−0.04 (0.24)	−0.26 (0.21)	0.04 (0.19)	−0.29 (0.22)
Size x Percent PLs		-	0.43 (0.20)*	0.38 (0.17)*	−0.56 (0.21)**
Random Intercept SD		0.62	0.52	0.44	0.55

*All level-2 variables are normalized to mean 0 and SD 1*.

+ *p < 0.10*,

* *p < 0.05*,

** *p < 0.01*.

**Table 4b:** 

**(B) Odds ratios from estimates in (a) with interactions evaluated at the mean**, −**1 SD, and** +**1 SD**				
	**Exposure modality**			
	**Presentation**	**Poster/video**	**Peer comm**.	**Activity**
**LEVEL-1**				
Gender	**0.83**	**0.56**	**0.56**	**0.65**
Ethnicity	0.86	1.00	0.97	1.00
Out-degree	1.03	1.00	0.97	1.01
Coreness	0.98	1.03	0.92	0.96
Closeness to PL	**1.13**	**1.25**	-	**1.15**
−1 SD coreness	-	-	**1.40**	-
Mean coreness	-	-	**1.54**	-
+1 SD coreness	-	-	**1.68**	-
Trusted adults	**1.08**	**1.09**	**1.16**	**1.11**
Suicide attempt	0.76	**0.69**	0.98	0.84
Suicide ideation	0.91	0.93	0.93	0.91
**LEVEL-2**				
Density	0.82	0.80	0.76	1.18
Percent PLs	1.18	-	-	-
−1 SD size	-	1.41	1.35	0.99
Mean size	-	**2.17**	**1.97**	0.57
+1 SD size	-	**3.34**	**2.87**	**0.32**
Size	0.96	-	-	-

*p < 0.05 in bold*.

Percent of students trained as a peer leader (a level-2 variable) still predicted poster/video exposure and direct peer communication with student closeness to a peer leader in the model (noting that percent of students trained as peer leaders was highly correlated with schoolwide closeness to a peer leader). The effect of schoolwide percent of students trained as peer leaders on exposure varied by school size for all exposures other than presentation. The effect of percent students trained as peer leader was greater for larger schools on poster/video and peer communication (interaction logit ranged from 0.38 to 0.43, *p's* < 0.05). Evaluated at the mean of school size, a one standard deviation increase in the percent of students trained as peer leaders was associated with a 2.17 likelihood of having seen a poster/video and a 1.97 likelihood of having direct peer communication (*p* < 0.05). This effect was greater in magnitude and significant for larger schools (+1 SD size) and nonsignificant for smaller schools (−1 SD size). Unexpectedly, this interaction was also significant for activity participation but the effects were in the opposite direction (interaction logit = −0.56, *p* = 0.007). The effect of schoolwide percent students trained as peer leaders was nonsignificant for schools of mean size or less, but was significant for larger schools (OR = 0.32, *p* = 0.04). To determine if this was an artifact of covariates an additional analysis was performed: school-level activity participation rate was regressed on school size, percent students trained as peer leaders, and their interaction. In this school-level OLS regression model (*N* = 20), the interaction term was marginally significant (B = −0.08, *p* = 0.08).

We included suicide attempt and suicide ideation-only individually into each of these models as interaction terms. There was no evidence that the individual and school predictors had a different impact on exposure for suicidal students, as shown by no significant interaction of suicide attempt or suicide ideation with any of the predictors (*p's* > 0.05).

## Discussion

After 1 year of Sources of Strength implementation in 20 high schools, exposure to the intervention varied widely across schools and as a function of individual student characteristics. Our findings regarding predictors of exposure build upon previous work on peer leader intervention diffusion by examining several indicators of students' connections to peers as well as adults. We found support for our hypothesis that students who were friends with peer leaders trained in Sources of Strength would have greater exposure to the intervention, across all messaging modalities. Students with peer leader friends had greater exposure to poster/video presentations, were more likely to have had direct communication about Sources of Strength from another student, and were more likely to have participated in an activity. These findings are consistent with prior research on peer-led programs (16–18) and with theoretical models (30, 41) that emphasize the importance of natural networks and direct personal affiliations as the medium through which peer leader prevention efforts are disseminated.

In addition to direct friendship ties with student peer leaders, our findings also showed that students who were closer in steps away from a peer leader in the friendship network also had greater intervention exposure. This suggests that peer leaders' social influence extends beyond their immediate friendship ties. Having a friend who is a friend of a peer leader may increase the social value of participating in Sources of Strength and attending to the intervention messaging. Interestingly, this effect was seen even for the exposure modalities such as posters that rely less on peer influence (e.g., students have an equal opportunity to see posters hung publicly). Having a friend or a friend of a friend who is a peer leader may make the intervention more salient in students' minds if, for example, they know they have friends involved in the program or they see a poster depicting a peer they know.

We found mixed support for our hypothesis that students with more overall friendship ties would have greater exposure to the intervention, as a function of having more social opportunities for new information. In univariate analyses, having more friends was modestly associated with intervention exposure, but this effect was not significant in the multivariate model that included closeness to peer leaders and other student and school characteristics. However, the effect of closeness to peer leaders on increasing exposure to direct peer communication was greater when students were part of a denser, more cohesive friendship network. This may arise as students in more dense friendship groups are more likely to have several friendship paths to a peer leader in contrast to a peer with a sparse friendship group.

**Figure 4:**
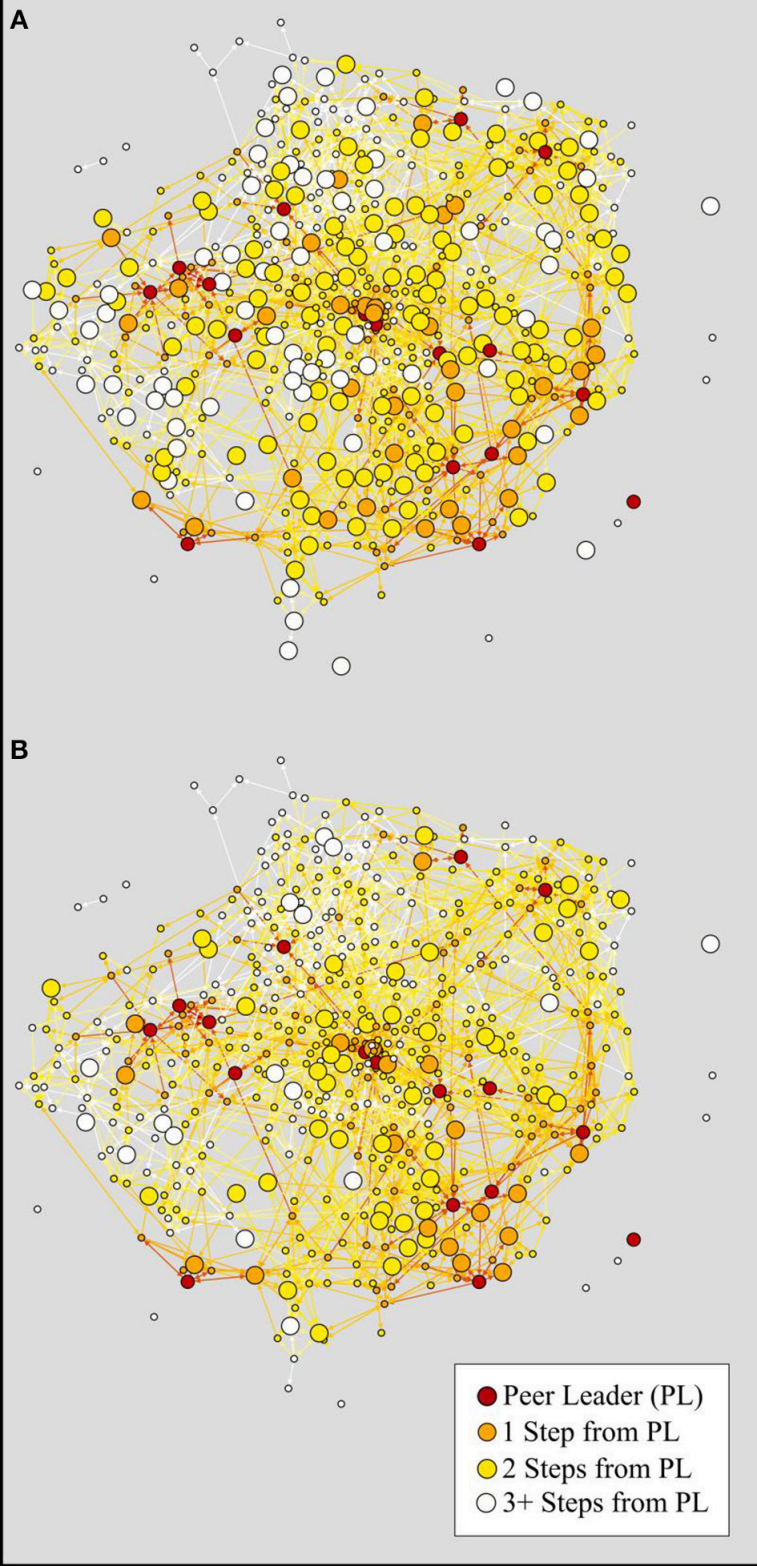
Comparison of two Sources of Strength exposures in a large school with few peer leaders. Nodes are colored by the distance to a peer leader. Large nodes have had exposure to the intervention through either a presentation **(A)** or direct peer communication **(B)**. This school has one of the lowest percentages of students trained as peer leaders (3.2%) and consequently has a low peer communication rate (20.3%) but a modest presentation exposure (53.7%).

As expected, the relationship between closeness to a peer leader and exposure varied in strength based on messaging modality. Being close to a peer leader was more beneficial for exposure to a poster/video and direct peer communication, and less so for presentation and activity participation. Figure 4 illustrates this finding with one of the largest schools in the study. This school—with low closeness to a peer leader and low percent of students trained as peer leaders—has one of the lowest peer communication rates of all schools in the study (Figure 4B). However, when examining the presentation exposure modality, which relies less on social connectedness, this school nonetheless has a modest proportion of students who have viewed a presentation (Figure 4A). While presentations may not have the ability to influence students' opinions as much as direct peer communication from a friend, they may have utility to disseminate awareness about the program to students in larger schools.

Our findings have especially relevant intervention implications for larger schools. We generally found that the greatest increases in intervention exposure occurred as the percent of students trained as peer leaders increased up to about 15% of the student population (after which the effect appeared to level off), a finding consistent with other studies (43, 55). Training this many peer leaders may be a more daunting task in larger schools, which we found generally had a lower proportion of students trained as peer leaders. In addition to logistical challenges posed when training more peer leaders, larger communities tend to form distinct sub-communities of segmented friendship groups which may hinder diffusion from the outside (56). Therefore, in larger schools it may be especially important to have more informed peer leader selection in order to distribute them in strategic areas of the network, thus using limited resources more effectively.

We also found that training more of the student population as peer leaders led to greater exposure to a poster/video and direct peer communication in medium to large schools (i.e., >150 students), adjusting for closeness to a peer leader. This finding—having an additional effect of peer leaders that doesn't act through closeness of direct friendship ties—may in part be due to the strength of so-called weak ties (i.e., acquaintances). These acquaintances are not typically captured by friendship surveys but have the ability to connect clusters of tightly-knit friendship groups and introduce new information to these social circles (57). The influence peer leaders exert on students may be largely explained by friendship ties in smaller, cohesive schools. At larger schools, though, training additional peer leaders could lead to increased opportunities for intervention exposure for individuals who are simply acquainted with these peer leaders (e.g., a peer leader taking the initiative to talk to a classmate). Indeed, weak ties' influence is stronger for message exposure than for behavior adoption, which may explain why this effect is not present for activity participation.

**Figure 5:**
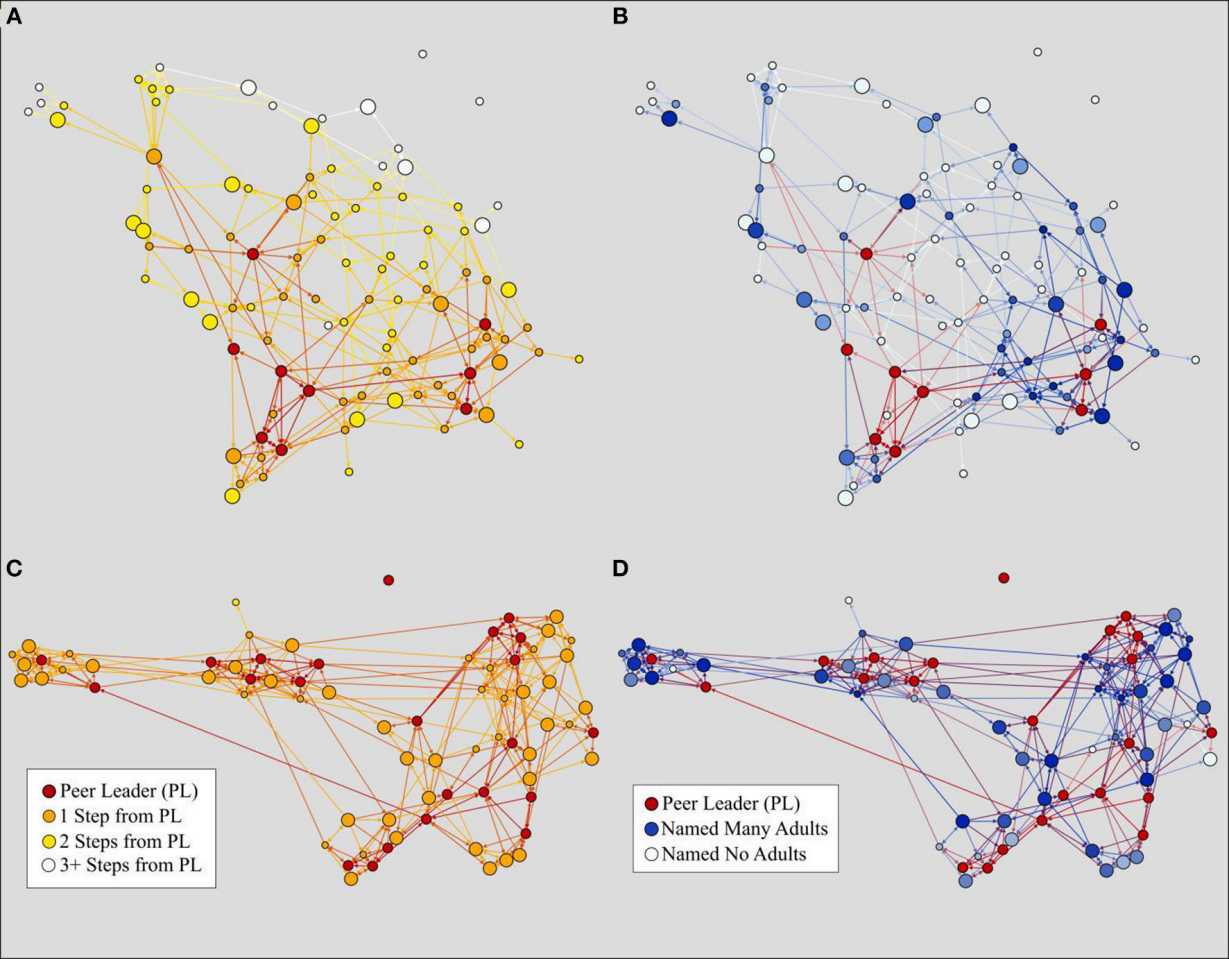
Two networks from schools of comparable sizes. Nodes are colored by the distance to a peer leader **(A,C)** or by number of trusted adults named **(B,D)**. Large nodes have had direct peer communication while small nodes have not. School A is size 103 with 8.7% peer leaders and 52.0% peer communication exposure, while School B is size 73 with 31.3% peer leaders and 73.5% peer communication exposure.

Closeness to a peer leader was one of the strongest predictors of intervention exposure, but there may be different ways to achieve this closeness as several network variables were correlated with the measure of closeness. Figure 5 illustrates two schools that are similar in size but have differing proportions of peer leaders and differing peer leader placement. The school in Figures 5C,D has higher network density, more students with ties to adults, more peer leaders, and peer leaders that appear to be more evenly placed through the network in comparison to the school in Figures 5A,B; it subsequently has a higher peer communication exposure (73.5 vs. 52.0%). This work demonstrates that messaging exposure is the greatest when peer leaders can reach most students with the shortest distance. Future work should address what combinations of network approaches can most effectively increase schoolwide closeness to peer leaders, or find new ways of engaging students in schools with more limited numbers of peer leaders.

Special efforts may be needed to reach male students and higher-risk youth than are currently being used in Sources of Strength. Male students in the study had consistently lower exposure to the intervention across modalities, even when males were directly tied by friendship to a student peer leader. Combined with our previous study showing that peer leader classroom messaging benefited females more than males, these findings indicate that different messaging strategies and content may be required to engage male students. Results from testing of tailored mental health promotion messaging to males in public service campaigns may be informative (58).

Likewise, we found that students with fewer connections to adults at school had consistently less exposure to the intervention across modalities. This gap is concerning given that absence of adult ties is a risk factor for a range of emotional and behavioral problems (59, 60). Additional methods of engaging youth to connect with adults at school—such as through text messaging—may provide unexplored opportunities for creating youth-adult communication and bonds.

Another at-risk group, students with suicide attempt, had modestly lower exposure to poster/video and presentation modalities, but had similar exposure to peer communication. This may suggest that students with suicide attempts are less engaged with regard to the modalities that rely less on interpersonal communication, perhaps due to other emotional or cognitive demands. On the other hand, students with prior suicidal behavior were found to engage in peer communication with others—a possible indicator of the effectiveness of the intervention's messaging activities in reaching this important sub-population.

Our finding that students' network characteristics increased intervention exposure similarly for suicidal and non-suicidal youth is promising (i.e., absence of a moderating effect of suicide attempt or ideation). This suggests that techniques used to increase exposure to the intervention (e.g., by creating denser friendship groups, fostering ties to adults, choosing peer leaders strategically to maximize closeness) will be effective for suicidal students as well as the general student population. More work is needed to clarify student attitudes—especially for higher-risk youth—and determine behaviors that appear to be discouraging their participation in the intervention.

### Strengths and limitations

We were able to examine the spread of the Sources of Strength intervention through 20 schools after 1 year. This study benefited from a large sample size, high response rates in each school, and being situated within a larger randomized controlled trial. Because the scope of this study is on dissemination of intervention messaging through the network, it is necessary to further determine how messaging exposure translates into behavior change and prevention effects.

There are some limitations to our study; namely, we cannot be sure that the intervention was spread only through peer-to-peer contact and we cannot be certain that the exposure questions assessed only messaging related to the Sources of Strength intervention. While it is reasonable to assume that messaging exposure is a key part of the intervention's impact, it is nonetheless possible that diffusion of the intervention may come about in other ways including social modeling of adaptive coping and help-seeking behaviors. And, while the student survey primed subjects to think about Sources of Strength, it is possible that some students may have provided false positive responses to exposure questions. For example, students may have found mental health information online, such as on YouTube, and reported that they had seen a video on suicide prevention even if that video was not a part of the Sources of Strength activities.

Sources of Strength was designed to be delivered over 2 years, yet our data come from the conclusion of the first school year. Within this 1-year period there may have been varying degrees of schoolwide and individual participation that could have influenced the effectiveness of the program. Future work should address how peer leaders' participation and engagement in delivering activities affects diffusion and the program's efficacy. Our findings should be interpreted cautiously, as it may take more time for peer leader training and messaging to reach its full effect in some schools.

We also found that, while percent of students trained as peer leaders was positively related to activity participation, there was a negative interaction with school size such that percent students trained as peer leaders led to lower activity participation in larger schools (>500 students). This may be due to an implementation issue where training more peer leaders exhausted resources that could have been devoted to planning activities. There was also less variation in the proportion of students trained as peer leaders in large schools; future work should attempt to replicate this effect with more variability in the proportion of students trained as peer leaders.

## Conclusion

In the first year of a 2-year intervention study, our findings show that peer leaders across 20 schools were able to disseminate the Sources of Strength intervention within a few months to substantial portions of their school population. Peer leaders reached students at high risk for suicide (due to past year suicide attempt), with regard to direct peer communication about Sources of Strength and through an interactive activity, similarly to other students. These findings suggest that a peer-led intervention may be an important complement to other intervention strategies designed at reaching higher-risk youth. Network information analyzed in this study underscores the challenges involved with reaching youth who are more disconnected from peer friendship networks and from adults at school. Future work with the intervention can take this information into account. One important priority is to determine how to leverage information on school friendship network structure to optimize how peer leaders are able to diffuse the intervention in schools with different patterns of connectedness.

## Author contributions

PW originated the study and supervised all aspects of its implementation, reviewed data analyses, and co-led writing of the article. TP conducted all network data analyses and co-led writing of the article. KS-C and CH conducted descriptive analyses and contributed to the writing of the article. TV, AP, and CB contributed to study design, reviewed analyses and contributed to writing of the article. KR contributed to interpretation of the findings and contributed to the writing of the article. ML originated the intervention and led the implementation of the intervention.

### Conflict of interest statement

ML is Executive Director and owner of Sources of Strength, Inc., which distributes the Sources of Strength intervention. The remaining authors declare that the research was conducted in the absence of any commercial or financial relationships that could be construed as a potential conflict of interest.
